# Administration Timing of Respiratory Syncytial Virus Preventatives Among Commercially Insured Populations in the United States: 2024–2025 RSV Season

**DOI:** 10.3390/vaccines14060471

**Published:** 2026-05-25

**Authors:** Amy W. Law, Danielle C. Mayer, Marjan Zakeri, Nehir Yapar, Alexandra Passarelli, Onur Baser, Pia D. M. MacDonald

**Affiliations:** 1 Pfizer, Inc., New York, NY 10001, USA; pia.macdonald@pfizer.com; 2 Pfizer, Inc., Collegeville, PA 19426, USA; danielle.mayer@pfizer.com; 3 Columbia Data Analytics, New York, NY 10013, USA; marjan@cdanyc.com (M.Z.); nehir@cdanyc.com (N.Y.); onur.baser@sph.cuny.edu (O.B.); 4 Graduate School of Public Health, City University of New York (CUNY), New York, NY 10027, USA

**Keywords:** respiratory syncytial virus, maternal vaccination, infant, administration timing

## Abstract

**Background/Objectives:** Respiratory syncytial virus (RSV) is the leading cause of infant hospitalizations in the United States. Prevention strategies are recommended to mitigate severe RSV outcomes. In addition to identifying potential coverage gaps, preventative administration timing is important for estimating product effectiveness. This study characterized administration timing of maternal and infant immunization against RSV across the United States during the 2024–2025 RSV season. **Methods:** A retrospective cross-sectional study was conducted using administrative claims of a commercially insured population from Kythera Labs. Pregnant individuals who received RSVpreF vaccine and infants who received nirsevimab were included. The seasonal cohort included infants born during the RSV season, while infants born from April to September were considered as the catch-up cohort. Baseline characteristics and calendar month and age at immunization (gestational age for RSVpreF) were evaluated. **Results:** Overall, 37,686 (71.9%) of maternal vaccinations were administered at 32–34 gestational weeks and 92.7% of all vaccinations occurred ≥14 days before delivery. Among infants who received nirsevimab, 34.8% of the seasonal cohort were immunized within 1 week of birth and 33.4% of the catch-up cohort were immunized in October 2024. **Conclusions:** Most maternal RSVpreF vaccinations occurred early in the recommended eligible gestational age window, while only approximately one-third of infants received nirsevimab during the first week of life or at the beginning of the RSV season. These findings highlight the importance of timely administration of RSV preventives. They further demonstrate that immunization timing should be incorporated into evaluation of the effectiveness and population level impact of RSV prevention programs.

## 1. Introduction

Respiratory syncytial virus (RSV) remains the leading cause of lower respiratory tract disease and hospitalizations among infants in the United States, with hospitalization rates reaching 23.8 per 1000 during the first 2 months after birth [1,2,3,4]. In 2023, two preventive interventions were approved in the United States to help protect infants from RSV-related lower respiratory tract disease (RSV-LRTD): a bivalent RSV prefusion F protein unadjuvanted vaccine (RSVpreF) administered to pregnant individuals as a single dose to provide passive immunity to infants and nirsevimab, a long-acting monoclonal antibody administered directly to infants [5,6]. The Advisory Committee on Immunization Practices (ACIP) recommends specific administration windows for RSV preventives [5,6]. Maternal RSVpreF is recommended for pregnant individuals between 32^0/7^ and 36^6/7^ weeks of gestational age (wGA) from September through January for most of the continental United States. Nirsevimab is recommended for infants <8 months of age born during or entering their first RSV season and for high-risk infants 8–19 months entering their second RSV season; optimal administration timing is within 1 week after birth but ideally occurs during the birth hospitalization, for infants born during the RSV season [7].

Due to the varying risk of RSV disease depending on infant age [8] and RSV seasonality [9], a granular evaluation of the administration timing of RSV preventions is important in identifying potential gaps in protection. Moreover, properly accounting of administration timing is necessary to understand peak antibody concentrations, thereby achieving optimal effectiveness. Xu et al. reported that infants require ≥7 days post-nirsevimab immunization to achieve peak serum antibody concentrations [10], whereas for RSVpreF maternal vaccine, immunogenicity data indicated that neutralizing antibody titer levels among infants were higher with longer gaps of >14 days between vaccination and delivery [11,12]. Together, the specific timing of administration plays an important role in calibrating the starting point for measuring the impact of immunization and comparing product effectiveness.

Select uptake timing parameters for these preventive interventions have been described for the 2023–2024 season [13] and for part of the 2024–2025 RSV season [14]. Given potential rollout and supply shortage issues related to the first RSV season when these RSV preventives were available as well as incomplete information for the second season, this study aimed to describe the real-world administration timing of RSVpreF vaccination among pregnant individuals and nirsevimab among infants during their first RSV season in the United States using complete data for the 2024–2025 RSV season.

## 2. Materials and Methods

### 2.1. Study Design and Data Source

This retrospective cross-sectional study utilized closed administrative claims data from Kythera Labs among the commercially insured population in the United States. The Kythera Labs database captures comprehensive medical and pharmacy claims for members, including 172 million individuals with commercial insurance across the United States [15]. De-identified patient records include details such as age, sex, insurance type, ZIP code, diagnoses, procedures and medications, with monthly updates ensuring recency and reliability [15]. The study focused on administration of preventative products to protect infants in the 2024–2025 RSV season (i.e., 1 October 2024–31 March 2025), the first full season where both the RSVpreF vaccine for pregnant individuals and nirsevimab immunization for infants were widely available.

### 2.2. Study Populations

Two distinct study populations were evaluated: a pregnant cohort who received the RSVpreF maternal vaccine and an infant cohort who received nirsevimab immunization in accordance with the recommended schedule.

Pregnant individuals were included if they (1) delivered a full-term (≥37 wGA) live-born infant between 1 September 2024 and 31 March 2025; (2) had sufficient data to apply the Moll et al. pregnancy outcome algorithm for gestational age estimation [16]; (3) had continuous commercial insurance enrollment from the estimated start of the pregnancy to the delivery date, allowing for a maximum 30-day gap in coverage within the same health plan; (4) were aged ≥18 years; and (5) received the RSVpreF maternal vaccine between 32^0/7^ and 36^6/7^ gestational weeks from 1 September 2024 to 31 January 2025.

Infants were included if they (1) were born between 1 April 2024 and 31 March 2025; (2) had continuous commercial health plan enrollment from birth through the end of the study (31 March 2025) or healthcare coverage termination, allowing for a maximum 30-day gap in coverage, whichever occurred first; and (3) had at least 1 claim for nirsevimab between 1 October 2024 and 31 March 2025. Infants were excluded if they received palivizumab after birth through 31 March 2025.

Infants immunized with nirsevimab were classified into two cohorts based on birth timing: a seasonal cohort for infants born during the RSV season (1 October 2024–31 March 2025) and a catch-up cohort for infants born outside of the RSV season (1 April 2024–30 September 2024) and <8 months when entering their first RSV season.

### 2.3. Demographic and Clinical Characteristics

Maternal demographic characteristics measured at delivery included maternal age (continuous and categorical: 18–25, 26–30, 31–35, 36–40, 41–45, 46–49 years), US geographic region (Northeast, Midwest, South, West), and socioeconomic status quintiles. Socioeconomic status was derived using a summary measure adapted from methods by Diez Roux et al. [16] to incorporate census-based data on income, education, and occupation linked to patient ZIP codes. Pregnancy characteristics included gestational week at delivery, administration of tetanus, diphtheria, and acellular pertussis (Tdap), influenza, and COVID-19 vaccines, and number of antenatal visits.

Infant characteristics measured at birth included sex, geographic region, socioeconomic status quintiles, birth status (full-term ≥37 weeks vs. preterm <37 weeks), birth hospitalization length of stay (LOS), birth weight categories (Supplementary Table S1), and underlying medical conditions (e.g., bronchopulmonary dysplasia, cystic fibrosis, congenital heart disease) diagnosed (Supplementary Table S2) between the date of birth and the date of nirsevimab immunization.

### 2.4. Study Measures and Analysis

RSVpreF administration timing was determined based on wGA and calendar month of vaccination. Gestational age referred to the gestational week at which the vaccine was administered. Gestational week was determined by applying the Moll et al. algorithm to first estimate the start of pregnancy and then calculating the wGA at vaccination in increments of 7 days from the start of pregnancy [17]. Timing was assessed between 32^0/7^ and 36^6/7^ weeks of gestation up to 1 day before delivery.

Nirsevimab immunization timing was analyzed by infant age and by birth month for each cohort. For the seasonal cohort, who were eligible to receive nirsevimab during the RSV season, immunization was assessed by weekly increments since birth (e.g., <1 week, 1 to <2 weeks, etc.); for the catch-up cohort, who were eligible to receive the immunization at the beginning of the season (i.e., October), timing was assessed by month of receipt during the RSV season.

Numbers and percentages were provided for categorical variables, and means with standard deviations were provided for continuous variables. All analyses were performed using R version 4.4.x (R Foundation for Statistical Computing, Vienna, Austria) and PySpark version X.X (Apache Software Foundation, Forest Hill, MD, USA).

### 2.5. Ethics Statement

This study utilized de-identified secondary patient data and was fully compliant with the Health Insurance Portability and Accountability Act (HIPAA). As such, this study was exempt from ethics committee approval.

## 3. Results

### 3.1. Pregnant Population

**Figure 1:**
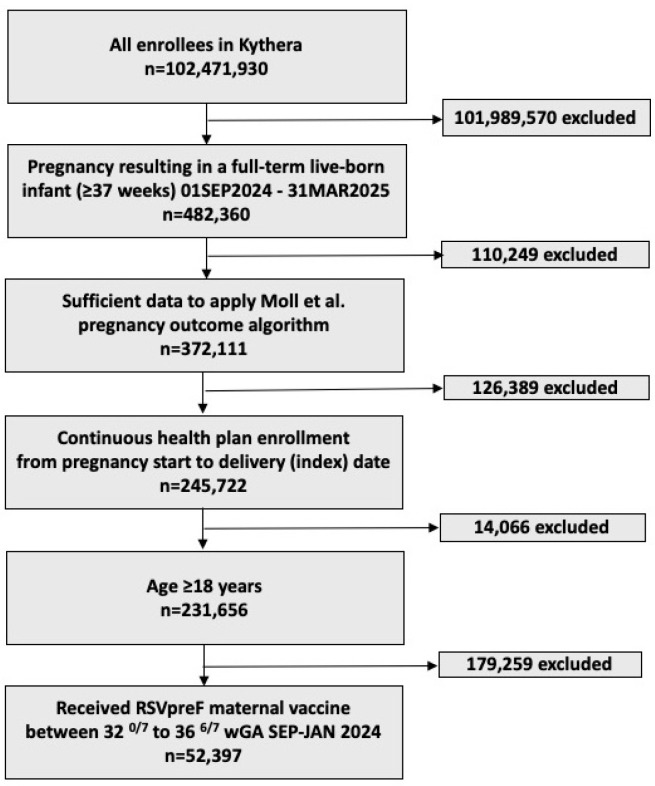
Attrition flowchart for the pregnant population, 1 September 2024–31 March 2025.

After applying selection criteria, the pregnant population included 52,397 individuals who received RSVpreF vaccination (Figure 1). The mean maternal age of those who received the vaccine was 31.3 years (SD = 5.3). The geographic distribution of the cohort included the South (28.6%), Midwest (27.2%), West (17.2%), and Northeast (11.6%) (Table 1). Most deliveries occurred between 38 and 39 wGA (33.8% and 27.7%, respectively). Additionally, 59.7% received the Tdap vaccine, 41.8% received the influenza vaccine, and 19.7% received the COVID-19 vaccine during pregnancy.

### 3.2. Immunized Infant Population

**Figure 2:**
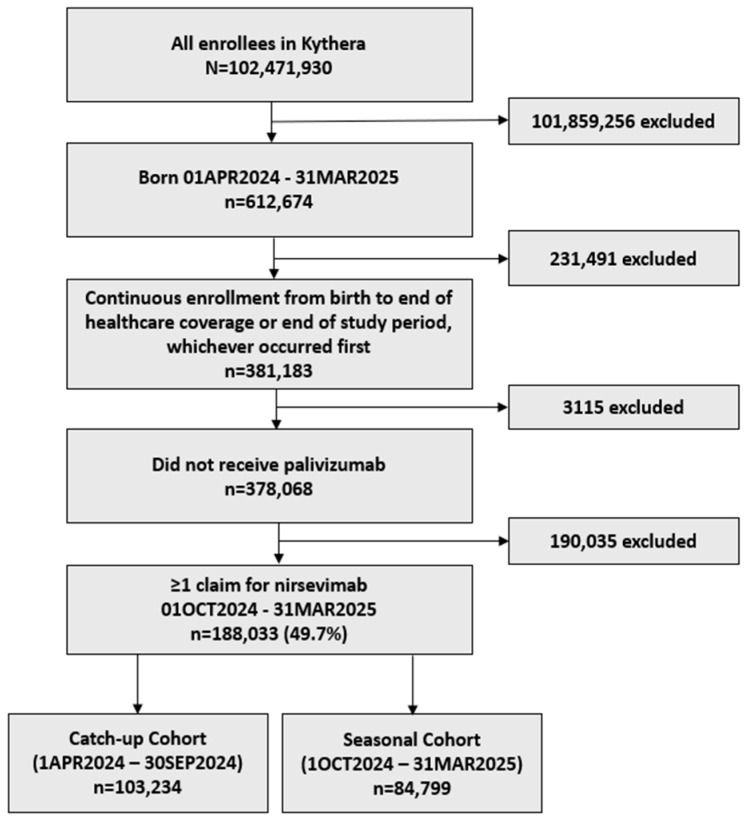
Attrition flowchart for the immunized infant population, 1 April 2024–31 March 2025.

A total of 188,033 infants received nirsevimab immunization during the 2024–2025 RSV season, including 84,799 (45.1%) infants in the seasonal cohort and 103,234 (54.9%) in the catch-up cohort (Figure 2).

Among the 188,033 infants who received nirsevimab immunization, the majority (98.6%) were born full-term, and the mean birth hospitalization LOS was 2.7 days (SD = 5.5; Table 2). A small proportion, 2.4%, of infants had underlying medical conditions, with congenital laryngeal stridor being the most common (1.4%). The infants in the seasonal and catch-up cohorts were similar in that the majority were born full-term, <3% in each cohort had any underlying condition, and both cohorts had a mean birth hospital LOS of less than 3 days (Table 2).

### 3.3. RSVpreF Administration Timing

**Figure 3:**
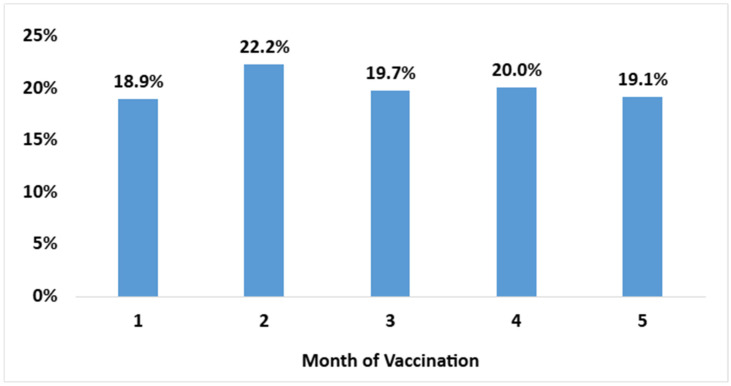
Month of RSVpreF vaccination, September 2024–January 2025 (N = 52,397).

**Figure 4:**
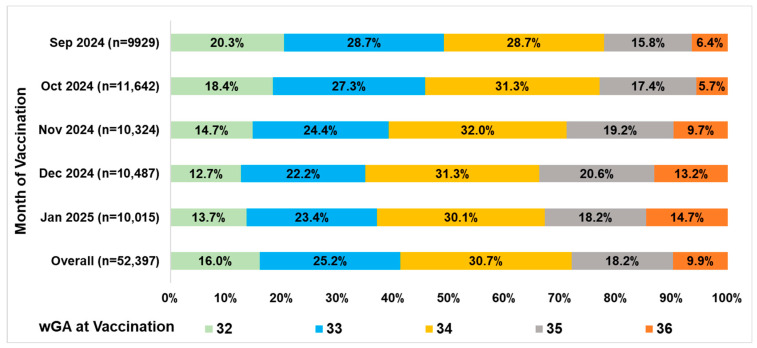
RSVpreF vaccination by wGA, September 2024–January 2025.

Across the recommended vaccination season from September through January, the monthly distribution of RSVpreF vaccination was relatively uniform, ranging from 18.9% to a peak of 22.2% during October (Figure 3). Most (92.7%) of the vaccinations were administered ≥14 days before delivery. Overall, 37,686 (71.9%) vaccinations were administered at 32–34 wGA (Figure 4). The distribution of vaccinations by gestational age was similar throughout the vaccination season. The largest proportion of vaccinations was administered to women at 34 wGA.

### 3.4. Nirsevimab Administration Timing

**Figure 5:**
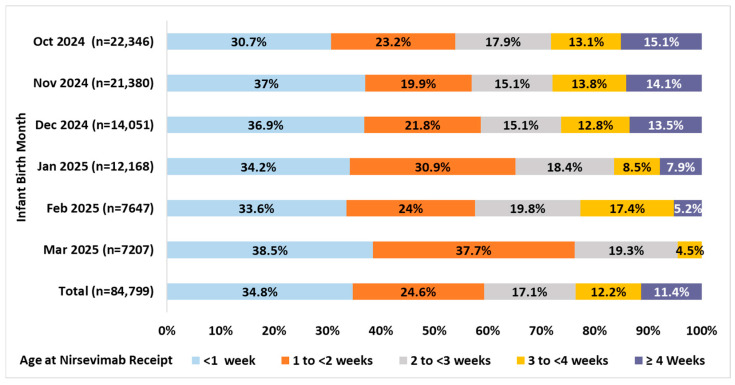
Age at Nirsevimab receipt during the RSV season by birth month in the seasonal cohort.

Among the 84,799 infants included in the seasonal cohort, 34.8% received nirsevimab during the first week of life, 24.6% at 1–<2 weeks, 17.1% at 2–<3 weeks, 12.2% at 3–<4 weeks, and 11.4% at ≥4 weeks (Figure 5). There were variations in immunization timing by birth month. Immunization during the first week of life was the lowest among infants born in October (30.7%) and highest among infants born in March (38.5%).

**Figure 6:**
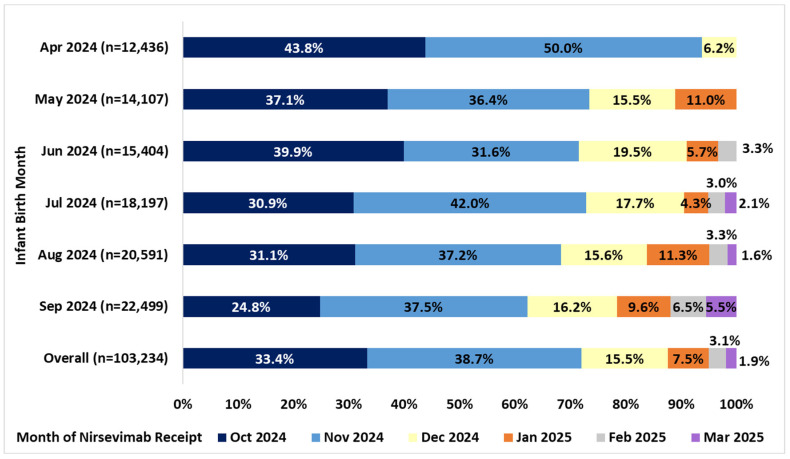
Month of nirsevimab receipt during the RSV season by birth month in the catch-up cohort.

Among the 103,234 infants in the catch-up cohort, 33.4% received nirsevimab in October, 38.7% in November, 15.5% in December, 7.5% in January, and 5% in February and March (Figure 6). Similar to the seasonal cohort, there were variations in immunization timing by birth month in the catch-up group. Approximately 43.8% of infants born in April received nirsevimab at the beginning of the season in October, while 24.8% of infants born in September received nirsevimab in October.

## 4. Discussion

This retrospective cross-sectional study of a US commercially insured population during the 2024–2025 RSV season found that among vaccinated pregnant persons, most RSVpreF vaccinations (71.9%) were administered during the first 3 weeks of the recommended gestational age window at 32 through 34 weeks, and 92.7% of vaccinations were administered ≥14 days before delivery. Among infants who were immunized with nirsevimab, approximately one out of three infants born during the season were immunized within the first week of life, and one out of three infants born off-season were immunized at the beginning of their first RSV season.

Our findings show relatively even RSVpreF maternal vaccination uptake across the recommended seasonality, with a peak in October. This pattern is similar to the CDC Vaccine Safety Datalink (VSD), which also showed an even distribution, with 19% of vaccinations in September, 18% in October, 25% in November, 16.9% in December, and 21% in January. Although the VSD peaked in November, both datasets suggest steady uptake throughout the season rather than concentration in a single month. Differences between our estimates and CDC figures likely reflect study methodology, including distinct data sources and selection criteria for the study population.

Our findings, of approximately one-third of infants from the seasonal cohort receiving nirsevimab within the first week of life, builds upon the 2023–2024 CDC data showing that 20.8% of doses were administered within 0–3 days and 17.2% within 4–6 days after birth (38% combined) [13]. Although only one third of infants in the catch-up cohort received nirsevimab by October, a majority of infants were immunized by November. Historically, RSV activity in the United States typically peaks in late December to January; thus, most infants in the cohort were immunized before substantial RSV circulation. However, it is important to note that RSV seasonality has been shifting in recent years. Earlier administration, particularly during the birth hospitalization or early in the season, may further optimize protection and reduce the window of vulnerability for infants born during the RSV season [7,18,19]. Lower proportions of infants receiving nirsevimab during the first week of life in the United States may reflect structural and implementation differences across healthcare systems. Part of the delay may be influenced by the hospitals’ participation in the Vaccines for Children (VFC) programs. Even though the VFC program only supports uninsured and underinsured children, which is not the population evaluated in this study, participation in such a program may have a broader effect on workflow that is not limited to the program-eligible children. Variability in hospital-based immunization infrastructure may also limit the ability to deliver nirsevimab prior to discharge. These system level factors may contribute to delayed administration in the U.S. context. Given that nirsevimab requires 7 days to reach maximum concentration, delayed administration may leave a meaningful proportion of infants unprotected during a critical early period of risk.

In the catch-up cohort, only about one-third of infants received nirsevimab in October 2024, with the lowest uptake among the youngest infants (September-born infants). These delays indicate that many infants entered the season without timely protection [20]. This is especially important given the highest incidence of severe RSV illness among the youngest age group of <6 months and especially among those 0–<2 months [10].

This retrospective cross-sectional, claims-based study has several limitations. Because claims data are collected for billing rather than research, the study is subject to coding errors, misclassification, and incomplete capture of clinical information. Findings are limited to a commercially insured population and may not be generalizable to uninsured individuals or those covered by other payer groups, such as Medicaid. Claims submitted outside billable systems, including those from public health programs, the Vaccines for Children Program, or out-of-pocket vaccination, may not be captured and could lead to an underestimation of true coverage. For the pregnant population, the analysis was restricted to on-label maternal vaccination timing between 32^0/7^ and 36^6/7^ weeks of gestation and to vaccinations administered between September and January; pregnant individuals immunized outside this window were not included. The cohort was limited to women with full-term liveborn deliveries, and gestational age was estimated using a claims-based algorithm that may vary by approximately seven days. For the infant population, only nirsevimab administrations occurring between October and March were included, consistent with recommended seasonal use. Infants vaccinated outside this period, including those born in off-season months who may have received nirsevimab prior to October, were not captured, which may underestimate uptake outside the recommended window.

In addition, maternal RSVpreF vaccination records and infant nirsevimab immunization records were not linkable in this dataset; therefore, the study could not assess whether specific population subgroups preferentially received maternal vaccination, infant immunization, or both. Future research should evaluate immunization patterns among pregnant individuals and infants vaccinated outside recommended schedules and assess maternal and infant characteristics associated with adherence to immunization recommendations.

## 5. Conclusions

Findings from this retrospective cross-sectional healthcare claims-based study close important data gaps related to the real-world timing of RSV preventative administration among immunized individuals within eligibility windows. Understanding specific implementation practices is critical for evaluating public health programs to ensure timely protection of infants against RSV during periods of highest risk. Maternal vaccination pattern by wGA was steady across the recommended season, with the overwhelming majority of vaccinations occurring ≥14 days before delivery, offering optimal antibody transfer. On the other hand, infant immunization patterns showed that while those born during the RSV season often did not receive an immunization within the first week of life, the majority were immunized during the first four weeks of life. These findings highlight the importance of timely administration and the need to account for immunization timing when evaluating the effectiveness and population impact of RSV prevention programs.

## Figures and Tables

**Table 1:** Baseline characteristics of the pregnant population vaccinated with RSVpreF between 1 September 2024 and 31 January 2025.

Characteristics	Vaccinated Cohort (N = 52,397), n (%)
Age, years	
Age, mean (SD)	31.3 (5.3)
18–25	7900 (15.1)
26–30	14,135 (27.0)
31–35	18,701 (35.7)
36–40	9831 (18.8)
41–45	1711 (3.3)
46–49	119 (0.2)
US geographic region *	
Northeast	6085 (11.6)
Midwest	14,266 (27.2)
South	14,967 (28.6)
West	9015 (17.2)
Other/Missing †	8064 (15.4)
Socioeconomic status	
Quintile 1 (Lowest)	8052 (15.4)
Quintile 2 (Low)	9516 (18.2)
Quintile 3 (Medium)	10,519 (20.1)
Quintile 4 (High)	10,989 (21.0)
Quintile 5 (Highest)	11,349 (21.7)
Unknown	1972 (3.8)
Gestational age at delivery, week	
37	13,882 (26.5%)
38	17,713 (33.8%)
39	14,499 (27.7%)
40	5076 (9.7%)
41	901 (1.7%)
≥42	326 (0.6%)
Maternal vaccinations administered during pregnancy	
Tdap	31,299 (59.7)
Influenza	21,881 (41.8)
COVID-19	10,314 (19.7)
Number of antenatal visits (mean ± SD)	10.3 ± 9

* Geographic regions follow the US Census Bureau regions. † “Other/Missing” refers to US territories and patients with missing geographic information. Abbreviation: SD, standard deviation.

**Table 2:** Baseline characteristics of the infant population who received nirsevimab between 1 October 2024 and 31 March 2025.

Characteristics	Immunized Infant Cohort (N = 188,033), n (%)	Seasonal Cohort * (N = 84,799), n (%)	Catch-up Cohort * (N = 103,234), n (%)
Sex			
Female	90,369 (48.1)	40,671 (48.0)	49,698 (48.1)
Male	93,876 (49.9)	42,419 (50.0)	51,457 (49.8)
Unknown/missing	3788 (2.0)	1709 (2.0)	2079 (2.0)
US Geographic region †			
North	33,120 (17.6)	12,853 (15.2)	20,267 (19.6)
Midwest	32,618 (17.3)	11,901 (14.0)	20,717 (20.1)
South	76,944 (40.9)	37,774 (44.5)	39,170 (37.9)
West	40,828 (21.7)	20,382 (24.0)	20,446 (19.8)
Other/Missing ‡	4523 (2.4)	1889 (2.2)	2634 (2.6)
Socioeconomic status			
Quintiles 1 (Lowest)	33,423 (17.8)	18,351 (21.6)	15,072 (14.6)
Quintiles 2 (Low)	35,920 (19.1)	17,309 (20.4)	18,611 (18.0)
Quintiles 3 (Medium)	36,871 (19.6)	16,440 (19.4)	20,431 (19.8)
Quintiles 4 (High)	39,236 (20.9)	16,715 (19.7)	22,521 (21.8)
Quintiles 5 (Highest)	39,367 (20.9)	14,739 (17.4)	24,628 (23.9)
Unknown/missing	3216 (1.7)	1245 (1.5)	1971 (1.9)
Birth status			
Full-term	185,468 (98.6)	83,586 (98.6)	101,882 (98.7)
Preterm	2565 (1.4)	1213 (1.4)	1352 (1.3)
Moderate-to-late preterm	2467 (1.3)	1146 (1.4)	1321 (1.3)
Early and extremely preterm	98 (0.1)	67 (0.1)	31 (0.03)
Birth length of stay (days) (mean ± SD) ^§^	2.7 ± 5.5	2.9 ± 3.4	2.7 ± 3.5
Birth weight categories			
Normal birth weight	186,663 (99.3)	84,041 (99.1)	102,622 (99.4)
Low birth weight	538 (0.3)	321 (0.4)	217 (0.2)
Very low birth weight	152 (0.1)	121 (0.1)	31 (0.03)
Moderately low birth weight	343 (0.2)	181 (0.2)	162 (0.2)
Exceptionally large newborn baby	26 (0.01)	9 (0.01)	17 (0.02)
Other heavy for gestational age	311 (0.2)	126 (0.2)	185 (0.2)
Underlying conditions ^¶^			
Bronchopulmonary dysplasia	57 (0.03)	41 (0.04)	16 (0.01)
Cystic fibrosis	310 (0.2)	175 (0.2)	135 (0.1)
Anatomic lung abnormalities	569 (0.3)	282 (0.3)	287 (0.3)
Congenital heart disease	142 (0.1)	87 (0.1)	55 (0.05)
Myoneural junction/muscle disorders	863 (0.5)	311 (0.4)	552 (0.5)
Congenital laryngeal stridor	2583 (1.4)	923 (1.1)	1660 (1.6)
HIV	5 (0.003)	1 (0.001)	4 (0.004)
Immunodeficiency	4 (0.002)	3 (0.003)	1 (0.0009)
Chromosomal abnormalities	334 (0.2)	169 (0.2)	165 (0.2)
Major organ transplant	6 (0.003)	6 (0.007)	0
Any of the above comorbidities	4482 (2.4)	1759 (2.1)	2723 (2.6)
Other comorbidities ^¶^			
Other respiratory-related conditions at birth	3766 (2)	1223 (1.4)	2543 (2.5)

* Catch-up cohort: infants born 1 April 2024–30 September 2024; Seasonal Cohort: infants born 1 October 2024–31 March 2025. † Based on US Census Bureau regions. ‡ ”Other/Missing” refers to US territories and patients with missing geographic information. ^§^ Infants without an inpatient discharge date or with emergency department-only records were excluded from mean and median length-of-stay calculations. ^¶^ Underlying and other conditions were measured prior to nirsevimab administration among infants. Abbreviation: SD, standard deviation.

## Data Availability

The data that support the findings of this study are available from Kythera Labs. Due to restrictions on the availability of these data, which were used under license for the current study, they are not publicly available. However, data may be made available from the corresponding author upon reasonable request and with the permission of Kythera Labs.

## Supplementary Materials

The following supporting information can be downloaded at: https://www.mdpi.com/article/10.3390/vaccines14060471/s1, Table S1. Infant Birth Weight ICD-10 Codes; Table S2. Infant Comorbidities.

## Abbreviations

The following abbreviations are used in this manuscript:

ACIP	Advisory Committee on Immunization Practices
CDC	Centers for Disease Control and Prevention
COVID-19	coronavirus disease 2019
HIPAA	Health Insurance Portability and Accountability Act
HIV	human immunodeficiency virus
LOS	length of stay
RSV	respiratory syncytial virus
RSV-LRTD	respiratory syncytial virus lower respiratory tract disease
RSVpreF	respiratory syncytial virus prefusion f protein vaccine
SD	standard deviation
Tdap	tetanus, diphtheria, and acellular pertussis (vaccine)
VSD	Vaccine Safety Datalink
wGA	weeks of gestational age

## References

[1] (2026). RSV in Infants and Young Children. Centers for Disease Control and Prevention. https://www.cdc.gov/rsv/infants-young-children/?CDC_AAref_Val=https://www.cdc.gov/rsv/high-risk/infants-young-children.html.

[2] Effectiveness and Impact of RSV Prevention Products in Infants During the 2024–2025 RSV Season. https://www.cdc.gov/acip/downloads/slides-2025-06-25-26/03-MacNeil-Mat-Peds-RSV-508.pdf.

[3] Suh M., Movva N., Jiang X., Reichert H., Bylsma L.C., Fryzek J.P., Nelson C.B. (2022). Respiratory Syncytial Virus Burden and Healthcare Utilization in United States Infants <1 Year of Age: Study of Nationally Representative Databases, 2011–2019. J. Infect. Dis..

[4] Curns A.T., Rha B., Lively J.Y., Sahni L.C., Englund J.A., Weinberg G.A., Halasa N.B., Staat M.A., Selvarangan R., Michaels M. (2024). Respiratory Syncytial Virus-Associated Hospitalizations Among Children <5 Years Old: 2016 to 2020. Pediatrics.

[5] RSV Vaccine Guidance for Pregnant Women. US Centers for Disease Control and Prevention. https://www.cdc.gov/rsv/hcp/vaccine-clinical-guidance/pregnant-people.html.

[6] Jones J.M., Fleming-Dutra K.E., Prill M.M., Roper L.E., Brooks O., Sánchez P.J., Kotton C.N., Mahon B.E., Meyer S., Long S.S. (2023). Use of Nirsevimab for the Prevention of Respiratory Syncytial Virus Disease Among Infants and Young Children: Recommendations of the Advisory Committee on Immunization Practices—United States, 2023. MMWR Morb. Mortal. Wkly. Rep..

[7] O’Leary S.T., Campbell J.D., Ardura M.I., Bryant K.A., Debiasi R.L., Espinosa C., Frenck R.W., Healy C.M., Herold B.C., John C.C. (2025). Recommendations for the Prevention of RSV Disease in Infants and Children: Policy Statement. Pediatrics.

[8] McMorrow M.L., Moline H.L., Toepfer A.P., Halasa N.B., Schuster J.E., Staat M.A., Williams J.V., Klein E.J., Weinberg G.A., Clopper B.R. (2024). Respiratory Syncytial Virus-Associated Hospitalizations in Children <5 Years: 2016–2022. Pediatrics.

[9] Jobe N.B., Rose E., Winn A.K., Goldstein L., Schneider Z.D., Silk B.J. (2025). Human Metapneumovirus Seasonality and Co-Circulation with Respiratory Syncytial Virus—United States, 2014–2024. MMWR Morb. Mortal. Wkly. Rep..

[10] Xu H., Aparicio C., Wats A., Araujo B.L., Pitzer V.E., Warren J.L., Shapiro E.D., Niccolai L.M., Weinberger D.M., Oliveira C.R. (2025). Estimated Effectiveness of Nirsevimab Against Respiratory Syncytial Virus. JAMA Netw. Open.

[11] Jasset O.J., Lopez Zapana P.A., Bahadir Z., Shook L., Dennis M., Gilbert E., Liu Z.A., Yinger R.V., Bald C., Bradford C.G. (2025). Enhanced placental antibody transfer efficiency with longer interval between maternal respiratory syncytial virus vaccination and birth. Am. J. Obstet. Gynecol..

[12] Simões E.A.F., Pahud B.A., Madhi S.A., Kampmann B., Shittu E., Radley D., Llapur C., Baker J., Pérez Marc G., Barnabas S.L. (2025). Efficacy, Safety, and Immunogenicity of the MATISSE (Maternal Immunization Study for Safety and Efficacy) Maternal Respiratory Syncytial Virus Prefusion F Protein Vaccine Trial. Obstet. Gynecol..

[13] Boundy E.O., Fast H., Jatlaoui T.C., Razzaghi H., Harris L., Nguyen K., Mells J., Peacock G., Black C.L. (2025). Respiratory Syncytial Virus Immunization Coverage Among Infants Through Receipt of Nirsevimab Monoclonal Antibody or Maternal Vaccination—United States, October 2023–March 2024; US Centers for Disease Control and Prevention. MMWR Morb. Mortal. Wkly. Rep..

[14] Implementation and Uptake of Nirsevimab and Maternal Vaccine for Infant Protection from RSV. National Center for Immunization and Respiratory Diseases. https://www.cdc.gov/acip/downloads/slides-2025-06-25-26/02-Peacock-Mat-Peds-RSV-508.pdf.

[15] Kythera Labs Kythera Labs, 2024. https://www.kytheralabs.com.

[16] Diez Roux A.V., Merkin S.S., Arnett D., Chambless L., Massing M., Nieto F.J., Sorlie P., Szklo M., Tyroler H.A., Watson R.L. (2001). Neighborhood of residence and incidence of coronary heart disease. N. Engl. J. Med..

[17] Moll K., Wong H.L., Fingar K., Hobbi S., Sheng M., Burrell T.A., Eckert L.O., Munoz F.M., Baer B., Shoaibi A. (2021). Validating Claims-Based Algorithms Determining Pregnancy Outcomes and Gestational Age Using a Linked Claims-Electronic Medical Record Database. Drug Saf..

[18] RSV Immunization Administration Frequently Asked Questions. American Academy of Pediatrics. https://www.aap.org/en/patient-care/respiratory-syncytial-virus-rsv-prevention/rsv-frequently-asked-questions/?srsltid=AfmBOopZ_l5L-xvhdruRLuU3gAWQwAHVQ66-jbqgnK9jNyQF93Q5ewfq.

[19] Olmsted K.E., Ramsey-Omonua T., Thomas E.S., Lee J.T., Owens L., Graitcer S., Mells J. (2025). Notes from the Field: Expanding Birthing Hospital Enrollment in the Vaccines for Children Program to Increase Infant Immunization Against Respiratory Syncytial Virus—United States, October 2023-March 2025. MMWR Morb. Mortal. Wkly. Rep..

[20] Griffin M.P., Yuan Y., Takas T., Domachowske J.B., Madhi S.A., Manzoni P., Simões E.A.F., Esser M.T., Khan A.A., Dubovsky F. (2020). Single-Dose Nirsevimab for Prevention of RSV in Preterm Infants. N. Engl. J. Med..

