# Supplementary material for: Administration Timing of Respiratory Syncytial Virus Preventatives Among Commercially Insured Populations in the United States: 2024–2025 RSV Season
Source: Vaccines (Basel). 2026 May 25;14(6):471. doi: 10.3390/vaccines14060471 (PMC13307636; doi:10.3390/vaccines14060471)
Supplement: Supplementary file 1 [file vaccines-14-00471-s001.zip › vaccines-4291915-supplementary.pdf]

Supplementary Materials

Table S1. Infant Birth Weight ICD-10 Codes

Birth Weight	ICD-10 CM Code(s)
Exceptionally large newborn baby	P08.0
Other heavy-for-gestational age newborn	P08.1
Low-birth-weight newborn (<2500 g) (ICD-10 code P07.0-P07.18)	P07.0-P07.18
Low-birth-weight newborns with unspecified weight	P07.00-P07.10
Very low birth weight (<1500 g)	P07.14-P07.15
Moderately low birth weight (1500–2499 g)	P07.18-P07.16

ICD-10, International Classification of Diseases, 10th Revision

Table S2. Infant Comorbidities

Comorbidities	ICD-10 Code(s)	CPT Code(s)	Algorithms
Bronchopulmonary dysplasia	P27*		≥1 Dx is in any position
Cystic fibrosis	E84*		
Anatomic lung abnormalities	J98.4, Q33.0, Q33.1, Q33.3, Q33.6		
Congenital heart disease	I42.9, I50.9, Q20.x, Q21.x, Q22.x, Q23.x, Q24.x, Q25.1, Q25.21, Q25.29, Q25.3, Q25.4x, Q25.5, Q25.6, Q26.0-Q26.4, Q26.8		
Myoneural junction and muscle disorders	G70-G73		
Congenital laryngeal stridor	P28.89		
HIV	B20, Z21		
Immunodeficiency	D80* D81*, D82*, D83*, D84*, D86*, D89*		
Chromosomal abnormalities	Q90*, Q99*		
Major organ transplant	Z94.81, Z94.82, Z94.83, Z94.84, Z94.89, Z94.0, Z94.1, Z94.2, Z94.4, Z94.9	38240, 38241, 33945, 33935, 47135, 50360, 50365	

Other respiratory-related conditions present at birth	J98.4, J95.821, J96.00, J96.01, J96.02, J96.90, P91.60, P22.0, P23.9, P25.0, P25.1, P25.2, P25.3, P25.8, P28.5, R91.8, J98.4, P23.x, P27.1, P27.9		

CPT, Current Procedural Terminology; Dx, diagnosis; ICD-10, International Classification of Diseases, 10th Revision
